# Isolation of Vascular Wall Mesenchymal Stem Cells from the Thoracic Aorta of Adult Göttingen Minipigs: A New Protocol for the Simultaneous Endothelial Cell Collection

**DOI:** 10.3390/ani13162601

**Published:** 2023-08-12

**Authors:** Chiara Bernardini, Debora La Mantia, Roberta Salaroli, Domenico Ventrella, Alberto Elmi, Augusta Zannoni, Monica Forni

**Affiliations:** 1 Department of Veterinary Medical Sciences, University of Bologna, Ozzano dell’Emilia, 40064 Bologna, Italy; chiara.bernardini5@unibo.it (C.B.); roberta.salaroli@unibo.it (R.S.); domenico.ventrella2@unibo.it (D.V.); alberto.elmi2@unibo.it (A.E.); augusta.zannoni@unibo.it (A.Z.); 2 Health Sciences and Technologies-Interdepartmental Center for Industrial Research (CIRI-SDV), Alma Mater Studiorum—University of Bologna, 40126 Bologna, Italy; monica.forni@unibo.it; 3 Department of Medical and Surgical Sciences, University of Bologna, 40138 Bologna, Italy

**Keywords:** vascular wall mesenchymal stem cells, large animal model, regenerative medicine, endothelial cells, miniature swine

## Abstract

**Simple Summary:**

It has been widely demonstrated that blood vessels are sources of multipotent progenitor cells, including mesenchymal stem cells. These stem cellular populations persist throughout adulthood and can be isolated from both microvascular and large vessels. Increasing evidence suggests that vascular stem cells, together with other cell populations residing in blood vessels, such as endothelial cells, are involved in physiological and pathological vascular remodeling. In the present paper, we described, for the first time, a new improved method to isolate a pure population of vascular wall cells showing a preserved mesenchymal tri-lineage differentiative potential from thoracic aorta of Göttingen Minipigs, preserving and also collecting endothelial cells. Considering the increasing interest in the use of Göttingen Minipigs as an animal model for cardiovascular diseases, the results obtained in the present research open the way to plan in vitro vascular remodeling experiments by using in co-culture system vascular mesenchymal stem cells and endothelial cells.

**Abstract:**

Two main classes of perivascular multipotent populations have been described: the microvascular pericytes and the vascular wall mesenchymal stem cells (VW-MSCs). VW-MSCs are isolated from large vessels in many species and they participate in vascular remodeling together with other cellular components such as endothelial cells. Considering that the Göttingen Minipigs are widely used in Europe as a translational model in the field of cardiovascular diseases, the aim of the present research was to isolate VW-MSCs from the adult aorta of Göttingen Minipigs while preserving and also collecting endothelial cells. The results obtained in the present research demonstrated that this new protocol allows us to obtain a pure population of VW-MSCs and endothelial cells. VW-MSCs from Göttingen Minipigs responded fully to the MSC minima international criteria, being positive to CD105, CD90, and CD44 and negative to CD45 and CD34. Moreover, VW-MSCs presented a differentiative potential towards osteogenic, chondrogenic, and adipogenic lineages. Overall, the present protocol, preserving the viability and phenotypic features of the two isolated populations, opens future possibilities of using minipig VW-MSCs and endothelial cells in in vitro vascular remodeling studies.

## 1. Introduction

It is now widely known that embryonic blood vessels are home to stem cells niches identified as natural precursors of mesenchymal stem cells; moreover, it has been demonstrated that these niches are also present in adulthood [1,2,3,4,5,6,7,8,9]. The microvascular pericytes [10,11] and the vascular wall mesenchymal stem cells (VW-MSCs) [12,13,14] are the two main classes of perivascular multipotent populations. A complex interaction between the progenitor cells and the terminally differentiated resident cells, like endothelial cells, occurs during post-natal life, concurrent with normal organ turnover but also with tissue pathologic remodeling, including inflammatory diseases, fibrosis and atherosclerosis [15,16,17,18]. VW-MSCs are isolated in large vessels in many species including humans [19], mice [20], rats [12] and bovines [2], and they are described as multipotent progenitors for postnatal vasculogenesis and angiogenesis [21]. In pigs, we isolated for the first time a population of VW-MSCs resident in the aorta of 3-month-old commercial hybrid pigs [22]. Porcine VW-MSCs (pVW-MSCs) cultured in vitro possess a typical MSC phenotype, maintain the osteogenic, chondrogenic and adipogenic trilineage potential and have the ability to differentiate in all the components of a mature vessel [23]. In addition, we have demonstrated that pVW-MSC secretome is enriched with bioactive factors, such as growth factors, cytokines and chemokines, that can modulate the immune response and promote angiogenesis [24,25].

In general, MSCs isolated from pigs represent an excellent translational model in the field of regenerative medicine due to the close similarity of pig MSCs to human ones [26,27,28,29]. Overall, the pig is a relevant animal model for preclinical studies using MSCs or MSC-derived infusions [30,31,32,33,34]. The possibility of creating in vitro models mimicking the angiogenic remodeling that occurs in vivo after tissue damage is certainly strategic for the success of regenerative medicine therapy [35]. In the last decade, miniature swine has been preferred to hybrid pigs for their small size, genetic stability and health status, making them a translational research model that is extremely helpful in biomedical research [36,37,38]. In particular, Göttingen Minipigs are widely used in Europe as a translational model in the field of cardiovascular diseases [39,40,41].

A deep phenotyping is increasingly needed for customized therapeutic approaches in the field of precision medicine, including for cardiovascular diseases [42]. Considering the influence of different cell populations on disease phenotype, it is highly relevant to set up method**s** to collect different cell populations from the same experimental animal to plan in vitro 3D heterotypic experiments.

By matching and optimizing two distinct protocols set up previously for the isolation of endothelial cells (pAECs, porcine aortic endothelial cells) [43] and VW-MSCs [22], the aim of the present research was to isolate both cell types from the adult aorta of a single Göttingen Minipig.

## 2. Materials and Methods

### 2.1. Chemicals and Reagents

Dulbecco phosphate-buffered saline (DPBS) with calcium and magnesium, Fetal Bovine Serum (FBS), trypsin-EDTA, antibiotic–antimycotic 100× (15240062), gentamicin, recombinant human epidermal growth factor (hEGF), Dulbecco’s Modified Eagle Medium: Nutrient Mixture F-12 (DMEM/F12), Hanks Balanced Salt Solution (HBSS) with calcium and magnesium, dimethyl sulfoxide (DMSO), diaminobenzidine, FluoroshieldTM with DAPI histology mounting medium, glucose and 2-[4-(2-hydroxyethyl) piperazin-1-yl] ethanesulfonic acid (HEPES), collagenase IA were purchased from Sigma-Aldrich (St. Louis, MO, USA). Betadine 10% cutaneous solution was purchased from Meda Pharma Spa (Milan, Italy). Endothelial Cell Medium (Human Endothelial SFM 11111044), DMEM, heat inactivated FBS, Trypsine 0.25%, StemPro Adipogenesis Differentiation Kit, the StemPro Osteogenesis Differentiation Kit, and the StemPro Chondrogenesis Differentiation Kit were purchased from Life Technologies (Carslab, CA, USA)). The Pericyte Growth Medium was purchased from PromoCell (Promocell, Heidelberg, Germany).

All plastic supports for primary cell culture were purchased from Corning–Beckton–Dickinson (Franklin Lakes, NJ, USA).

The antibodies used for the immunofluorescence (IF) and flow cytometry analyses (FC) are listed in Table 1 and were purchased from AbD Serotec (Kidlington, UK); Abcam (Cambridge, UK), Biolegend (San Diego, CA,USA) and Sigma-Aldrich (St. Louis, MO, USA).

### 2.2. Animal Description

To reduce the number of animals utilized, tissue samples were collected from animals enrolled in an experimental lactation study approved by the Italian Ministry of Health as dictated by D.Lgs 26/2014 (approval n° 32/2021-PR). All procedures were performed in compliance with ARRIVE guidelines. At the end of the experimental trial, sows, acquired by Ellegaard Göttingen Minipigs A/S (Dalmose, Denmark), were group housed with a light/dark cycle of 12:12 and hand fed with a standard commercial diet (Micropigs 9AB20; Mucedola srl, Settimo Milanese Italy). Pens were equipped with both chewable and wooden environmental enrichments. On the day of sacrifice, animals were sedated intramuscularly (IM) with 5 mg/kg of tiletamine/zolazepam (Zoletil, Virbac, Carros, France) and euthanized upon barbiturate overdose (Sodium thiopental 60 mg/kg; Pentothal sodium, MSD Animal Health srl, Madison, NJ, USA). Thoracic aortic traits (11 ± 2 cm; n = 3) were surgically removed and collected from 3 Göttingen Minipigs.

### 2.3. Histological Examination

Portions of 1 cm long portions from the aorta, pre and post enzymatic digestion, were embedded in OCT and frozen in isopentane cooled in liquid nitrogen. Ten-micrometer-thick sections were cut with a Leica CM1950cryostat (Leica, Wetzlar, Germany), then left to adhere to a microscope slide and stained with hematoxylin and eosin (H&E) according to the standard procedure. Images were obtained using a Nikon digital camera (DS-Qi2 Monochrome Digital Microscope Camera) installed on a Nikon epifluorescence microscope Eclipse E600 and analyzed with NIS-Elements BR Ver5.30.00 digital image software (Nikon, Tokyo, Japan).

### 2.4. Cell Isolation

**Figure 1:**
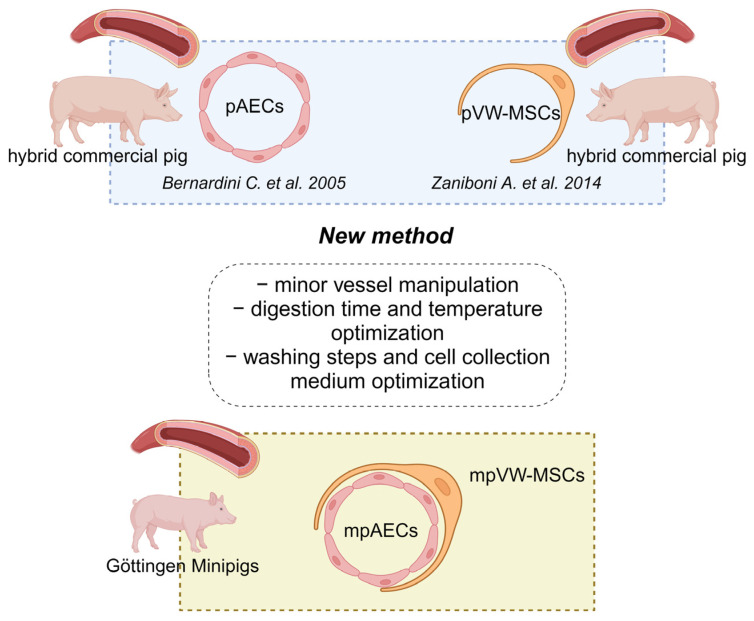
Summary scheme of the mpVW-MSCs and mpAECs isolation protocol. The image shows schematically the new protocol derived from two different methods, previously set up for the isolation of aortic endothelial cells and vascular wall mesenchymal stem cells obtained from hybrid commercial pigs. Minipig vascular wall mesenchymal stem cells (mpVW-MSCs); minipig aortic endothelial cells (mpAECs) (Created with BioRender.com, Agreement number: MR25PADPU8) [22,43].

pAEC and VW-MSC isolation from minipigs (mpAECs and mpVW-MSCs, respectively) were obtained by modifying two methods previously developed and described (Figure 1) for the isolation of porcine aortic endothelial cells (pAECs) and porcine vascular wall mesenchymal stem cells (pVW-MSCs) [22,43] from hybrid commercial pigs.

The explanted vessel was cleaned before being moved to the laboratory. Two additional washes were performed using Endothelial Medium plus 1× antibiotic–antimycotic (ECm 1% anti-anti), before transferring the vessel under a laminar flow hood.

All excess tissue was removed from the vessels and the aorta was cannulated. The lumen of the vessel was gently washed twice with ECm 1% anti-anti to remove the residual blood and then was filled with 0.2% collagenase IA solution and incubated at 38.5 °C. To recover the endothelial layer, collagenase solution containing cellular suspension was collected after 20 min of incubation in a sterile 50 mL tube, and the enzymatic reaction was stopped with an equal volume of 10% FBS. Then, the vessel was washed with 15 mL ECm (1% anti-anti) to collect the residual cell clusters. The cellular suspensions were centrifuged at 500× *g* for 10 min. The supernatant was discarded, and the cellular pellet was resuspended in ECm containing 10% FBS and 1× antibiotic–antimycotic solution and seeded in a 75 cm^2^ tissue culture flask (BD Primaria, BD Bioscience (Haryana, India)). The aorta was refilled with 0.2% collagenase IA solution and incubated for an additional 2 h at 38.5 °C. Collagenase solution containing cellular suspension was then recovered in a 50 mL tube, and the vessel was washed twice with DPBS and 1× antibiotic–antimycotic to collect the residual cell clusters. Collagenase enzymatic activity was then stopped by addition of an equal volume of 10% FBS. The cellular suspensions were centrifuged at 500× *g* for 10 min. The supernatant was discarded, and the pellet was resuspended in high glucose (hg) DMEM, to which a 10% FBS and 10× antibiotic–antimycotic solution (hgDMEM-10×) was added and seeded in a 75 cm^2^ tissue culture flask (BD Primaria, BD Bioscience).

### 2.5. Cell Expansion

mpAECs at the first passage were detached and expanded for at least three passages in EC medium containing 5% FBS and 1% antibiotic–antimycotic solution.

mpVW-MSCs at the first passage were detached and expanded in PGM medium to maintain their multipotent potential. Doubling times were calculated for both cell populations as indicated previously [44].

The freezing protocol for both mpAECs and mpVW-MSCs consisted of detaching cells at 70% of confluence using 0.25% Trypsin EDTA, washing them in DPBS, and counting and suspending them at a concentration of 0.5 × 10^6^ cells/mL in a freezing medium consisting of 10% dimethylsulfoxide (DMSO)+ 90% FBS. Cells were dispensed in cryovials tubes and were slowly cooled in a Mr. Frosty freezing container placed in a freezer at −80 °C before transferring to the nitrogen liquid biobank the following day.

### 2.6. Cell Characterization by Flow Cytometry Analysis

To confirm the endothelial or mesenchymal immunophenotype, flow cytometry analysis was performed. Briefly, 2 × 10^5^ cells were resuspended in 100 μL of phosphate-buffered saline (PBS) and incubated for 1 h at 4 °C in the dark with appropriate fluorochrome-conjugated antibodies at the titers reported in Table 1. Unstained controls, to evaluate the inherent background for autofluorescence, were obtained by omitting primary antibodies. After incubation, cells were washed twice and resuspended in 200 μL of PBS, then analyzed with MacsQuant Analyzer10 (Miltenyi Biotec, Bergisch Gladbach, Germany). For CD34 staining, after the first incubation with the primary antibody, cells were washed and incubated with PE-conjugated secondary antibody (Table 1) for 40 min at 4 °C in the dark. Data were analyzed using Flowlogic™ software (https://flowlogic.software/) (Inivai Technologies, Mentone, VIC, Australia).

### 2.7. mpAEC In Vitro Angiogenesis Assay

To verify the mpAECs ability to create a capillary-like network, an in vitro angiogenesis assay was carried out using 8-slide chambers (BD Falcon Bedford, MA, USA) coated with 100 μL of undiluted Geltrex™ LDEV-Free Reduced Growth Factor Basement Membrane Matrix (Thermo Fisher, Waltham, MA, USA). Extracellular matrix coating was carried out 1 h before the cell seeding in a humidified incubator at 38.5 °C and 5% CO_2_; then, 8 × 10^5^ mpAECs were seeded and incubated for 18 h. Images were acquired at 0, 3 and 18 h using a digital camera installed on a Nikon phase contrast microscope and analyzed by Image J 64 open software (National Institutes of Health, Bethesda, MD, USA). Then, mpAECs were gently washed with DPBS and fixed with 4% of paraformaldehyde, and immunohistochemistry staining for CD31 was performed as previously described [23].

### 2.8. mpVW-MSC Cell Adhesion Assay

To verify mpVW-MSCs ability to adhere to each other, forming a spheroid [45], the hanging drop method was used; 5 mL of DPBS was placed at the bottom of a Petri dish to create a hydration chamber, then drops of 6 × 10^4^ cells in 10 µL of PGM medium were seeded on the cover of the dish. Then, the cover was overturned and incubated at 5% CO_2_ and 38.5 °C. After 24 h, spheroids were checked for morphology and dimensions by an Eclipse E600 epifluorescence microscope equipped with a Nikon digital camera and ACT-2U software for image capturing (Nikon, Tokyo, Japan).

### 2.9. Mesenchymal Trilineage Differentiation Potential

To verify mpVW-MSCs ability to differentiate in the three mesenchymal lineage, mpVW-MSCs were cultured with a StemPro Adipogenesis Differentiation Kit, a StemPro Osteogenesis Differentiation Kit, a StemPro Chondrogenesis Differentiation Kit (all purchased from GIBCO-Life Technologies), or in standard culture conditions with PGM medium (control cells) for 21 days. After that, the cells were stained with OilRedO, Alizarin Red, and Alcian Blue (all purchased by Sigma-Aldrich) as indicated by the manufacturer’s protocol.

### 2.10. Statistical Analysis

Doubling time data were analyzed by a one-way analysis of variance (ANOVA) followed by a post hoc Tukey comparison test (*p* < 0.05) (GraphPad Prism 5 software). Flow cytometry data were analyzed with a Student’s *t* test comparing the MFI of the negative control and the MFI of single stained cells (*p* < 0.05) in each cell population.

## 3. Results

### 3.1. mpAECs and mpVW-MSCs Isolation

**Figure 2:**
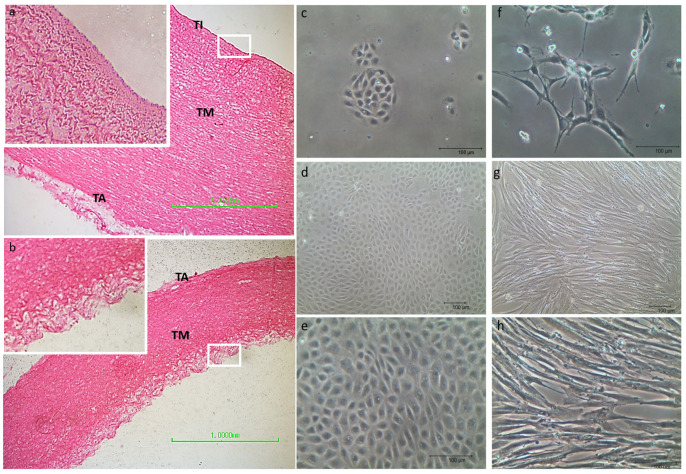
Representative images of thoracic aorta segments before enzymatic digestion with the typical three layers: the tunica intima (TI), the tunica media (TM), and the tunica adventitia (TA) (**a**); after two consecutive collagenase digestion treatments, the tunica intima was dissolved while the tunica media was partially digested (**b**); (**a**,**b**) scale bar = 1 mm. Representative images of small endothelial cellular islands after 24 h of isolation (**c**) and after reaching confluence (**d**,**e**). Representative images of VW-MSCs with elongated shape morphology at ~25% confluence (**f**) and the typical spindle shape fibroblast-like morphology at full confluence (**g**,**h**); scale bar 100 µm.

Sections from the minipig aorta wall before enzymatic digestion showed all the normal three layers: intima, media, and adventitia. The intima appeared as a flattened unicellular layer of endothelial cells (Figure 2a). After the two consecutive collagenase treatments, the endothelial cell layer, including the basal lamina, was detached and approximately one-sixth of the tunica media was digested (Figure 2b). After 24 h (Figure 2c), cells collected from the first digestion time (20 min) were attached to the plastic flask and they showed a typical endothelial morphology by growing flat in small island; after approximately 1 week, the cells reached the confluence, forming a compact monolayer (Figure 2d,e).

The cells collected after the second digestion time (2 h) and after the selective protocol described previously [22] displayed a stellate- or elongate-shaped morphology with centrally placed oval or round nuclei (Figure 2f); they grew slowly and reached confluence approximately after 3 weeks, showing a spindle shape fibroblast-like morphology (Figure 2g,h).

### 3.2. mpAEC and mpVW-MSC Characterization

**Figure 3:**
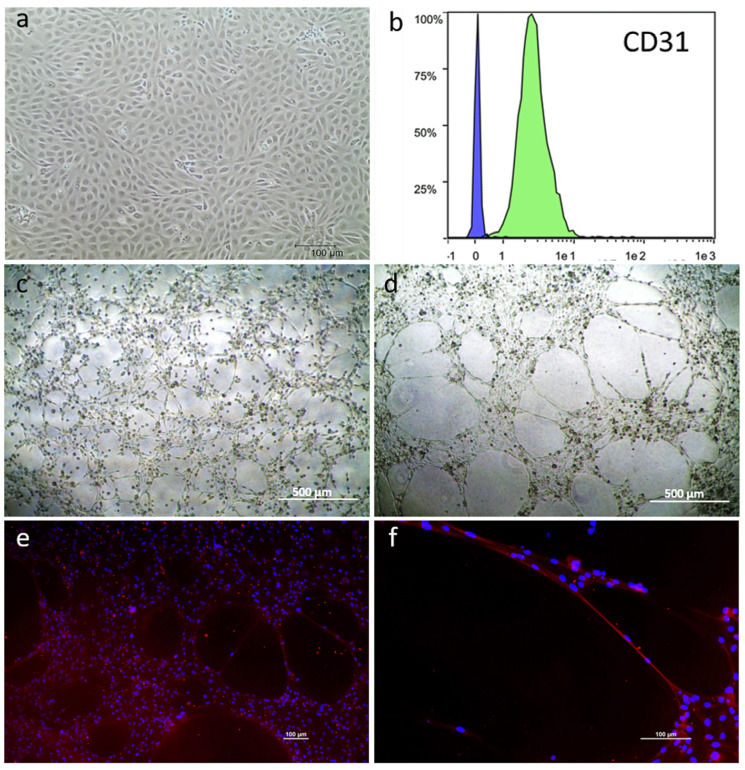
Representative images of mpAEC characterization: morphology (**a**) and cytometric analysis of CD31 marker: histogram shows the percentage of cells expressing or not CD31 in the green Area Under the Curve (AUC); the relative negative control consists of cells with no antibody incubation (blue AUC) (n = 3), (**b**), and the formation of a capillary-like endothelial network (**c**,**d**). Immunofluorescence analysis of VE-cadherin (**e**,**f**); nuclei are stained with DAPI (blue), (**a**,**e**,**f**) scale bar = 100 µm; (**c**,**d**) scale bar 500 µm.

The three primary mpAEC cell lines were successfully expanded in EC medium for three passages. They maintained the typical endothelial morphology (Figure 3a); the mean doubling time of the three primary cell lines was 35.30 ± 4.63 h. Cytometric analysis showed that all the cellular population were clearly positive to CD31 (Figure 3b) and cells seeded on an extracellular matrix showed the ability to create a capillary-like network. In particular, three hours after cell seeding, mpAECs were already aligned to form a network (Figure 3c). The capillary-like network was fully formed after 18 h (Figure 3d), and cells clearly expressed the endothelial marker VE-cadherin (Figure 3e,f). No differences were observed among the three primary cellular lines.

**Figure 4:**
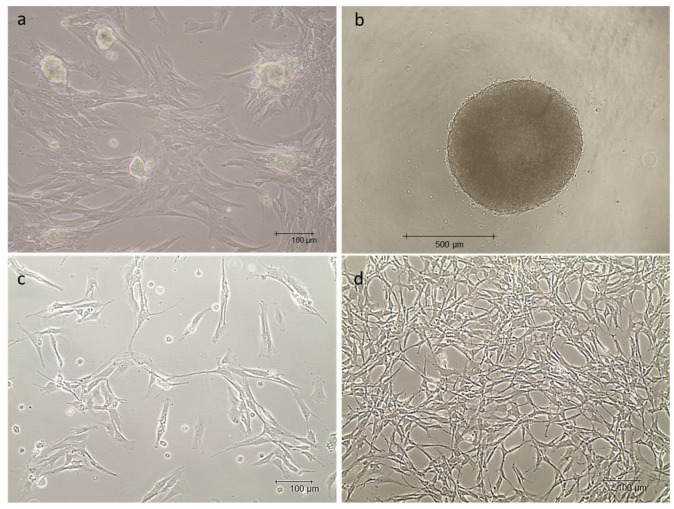
Representative images of mpVW-MSC morphology, showing the adhesive spindle shape cellular morphology with the formation of sporadic spheroidal structures, scale bar 100 µm (**a**); mpVW-MSC spheroid formation when cultured via the hanging drop method, scale bar 500 µm (**b**); mpVW-MSC morphology when the cells were grown in a PGM medium in sub-confluence and when reaching confluence (**c**,**d**), scale bar 100 µm.

The three primary mpVW-MSC lines cultured in no selective medium showed a typical spindle shape, fibroblast-like morphology and occasionally spherical formations sprouted from the cellular monolayer (Figure 4a). When cultured using the hanging drop technique, mpVW-MSCs were able to form compact spheroids with a mean diameter of 770.40 ± 69.79 µm (Figure 4b); no significant differences were observed among the three primary cellular lines. mpVW-MSCs expanded in PGM medium showed a typical perivascular mesenchymal cell morphology: a small cell body with thin and long cellular extensions (Figure 4c,d). No differences were observed among the three primary cellular lines (Figure S1).

**Figure 5:**
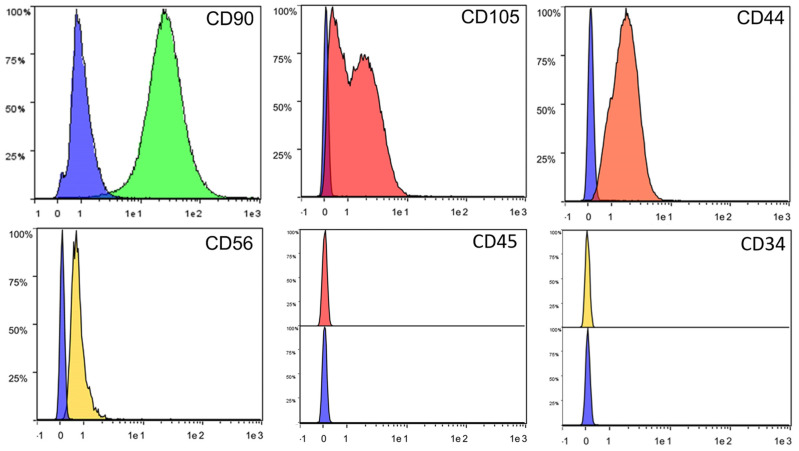
Representative image of immunophenotyping characterization of mpMVW-MSCs by a flow cytometry analysis. Each histogram shows the percentage of cells expressing or not markers in the Area Under the Curve (AUC); the relative negative control consists of cells with no antibody incubation (blue AUC), for CD45 and CD34, stacked histograms relative to positive and negative signals are shown (n = 3).

A flow cytometric analysis showed a typical MSC profile. In alignment with the criteria for MSC characterization, the cell populations were positive for the mesenchymal stemness markers CD105, CD90, and CD44 and for the specific vascular wall MSC marker CD56, whereas they were completely negative for the hematopoietic markers CD45 and CD34 (Figure 5).

### 3.3. mpVW-MSC Trilieaneage Potential

**Figure 6:**
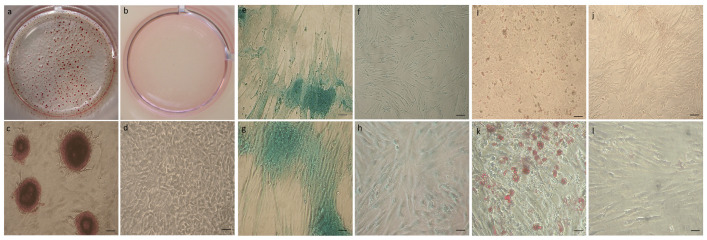
Representative images of mpVW-MSC osteogenic (**a**–**d**), chondrogenic (**e**–**h**) and adipogenic differentiation (**i**–**l**). After 21 days in specific pro-osteogenic medium, mpVW-MSCs formed bone enucleation aggregates positive to Alizarin red staining (**a**,**c**). Extracellular matrix production when cells were grown in pro-condrogenic medium (Alcian blue staining figure (**e**,**g**). Fat vesicle presence in mpVW-MSCs cultured in pro adipogenic medium (**i**,**k**). The negative control is represented by cells grown in standard culture conditions with PGM medium ((**b**,**d**) Alazarin red staining; (**f**,**h**) alcian blue staining, (**j**,**l**) Oil red staining). Scale bar (**e**,**f**,**i**,**j**) 100 µm; (**c**,**d**,**g**,**h**,**k**,**l**) 50 µm.

mpVW-MSCs were able to differentiate towards osteo, chondro, and adipocyte phenotypes. The cells displayed osteogenic (Alizarin Red stain Figure 6a,c), chondrogenic (Alcian Blue stain Figure 6e,g), and adipogenic (OilRedO stain Figure 6i,m) differentiation potentials after 21 days of treatment with differentiating culture media (Figure 6). Cells grown in pro-osteogenic medium for 21 days formed a typical center of bone enucleation that was positive to Alazarin red (Figure 6a,c); no positivity was observed in control cells (Figure 6b,d). mpVW-MSCs grown in pro-condrogenic medium clumped together, forming long bundles and secreting extracellular matrix, and were positive to Alcian Blue (Figure 6e,g). No positivity stain was observed in control cells (Figure 6f,h). pVW-MSCs grown in pro-adipogenic medium demonstrated the intercytoplasmic presence of fat vesicles that were positive for oil red staining (Figure 6i,k). No fat vesicles were observed in control cells (Figure 6j,l).

## 4. Discussion

Increasing studies indicate Göttingen Minipigs as an excellent translational model to study cardiovascular diseases [39,46,47,48,49,50], even in consideration of their propensity to develop various disorders of the metabolic syndrome, including obesity, insulin resistance, glucose intolerance, dyslipidemia, and atherosclerosis [39,51,52,53,54,55,56]. Clinical applications of mesenchymal stem-cell-based tissue engineering are promising due to the specific properties of these cells, i.e., high regenerative capacity, proliferative potential, immunomodulatory activity, and low immunogenicity [57]. Isolation of porcine MSCs from bone marrow (BM) was reported by several investigators [26,58,59,60]. Porcine MSCs were also isolated from various sources such as umbilical cord Wharton’s jelly [61,62], peripheral blood [63], amniotic fluid [64], umbilical cord blood (UCB) [65], adipose tissue [66], and the thoracic aortic wall [22]. The isolation of MSCs from the bone marrow of 14–16-month-old female Göttingen Minipigs was described for the first time in 2010 by Agay et al. [67]; then, they were also isolated from adipose tissue [68] and cardiac tissue [69].

The cross-talk between MSCs and endothelial cells in the vascular network is a known reality and the potential effects of this interaction are also well known [70]. In the presence of inflammatory stimuli, MSCs are able to increase the self-repair activity of endothelial cells, and this action appears to be carried out by increasing production of soluble factors; therefore, MSC transplantation is considered as a potential way to slow down the development of atherosclerosis [71]. Tissue engineering studies stress the need for autologous cell-based approaches, but clarification of processes that support the innate cellular capacity to remodel and form vasculature is still needed. In 3D eterotypic cultures, endothelial cell sprouting is dependent from MSC position in EC-MSC co-culture spheroids evidencing that the communication between different cell populations can define network formation [72].

In terms of the development of ex vivo and in vitro models, and to encourage the use of methods complementary to the in vivo ones, it could be of high interest to have cells of different types but belonging to the same subject, which could reconstitute in vitro multi-cellular models that can recapitulate the animal from which they were derived.

In the present study, we reported the simultaneous isolation of vascular wall mesenchymal stem cells and endothelial cells from the thoracic aorta of adult Göttingen Minipigs. The protocol described in the present research was developed from two distinct protocols for the isolation of endothelial cells [43] and VW-MSCs from the thoracic aorta of hybrid commercial pigs [22]. The new optimized protocol allows to isolate mpVW-MSCs resident in the first layers of the tunica media, as described for those cells isolated from commercial hybrid pigs [22]. mpMSCs showed a fibroblast-like morphology when cultured with a non-selective medium, as described by the International Society for Cellular Therapy [73] concerning the minimal criteria for defining multipotent mesenchymal stromal cells. Occasionally, in the first stage of growth, spheroid cellular formations spontaneously sprouted; the ability to form compact spheroids is a well-demonstrated ability of MSCs grown in particular conditions of no attached culture. In the present research, the mpVW-MSC’s ability to grow in a spheroid structure was confirmed when cells were cultured by the hanging drop technique. However, when mpVW-MSCs were cultured in a specific medium for multipotent pericytes, the cell morphology changed towards a phenotype characteristic of perivascular cells with a small cell body, thin and long cellular extensions [23]. A flow cytometric analysis showed a typical MSC profile in line with the criteria for vascular MSC characterization and in agreement with those observed in hybrid commercial pigs. Overall, the results obtained demonstrated that the protocol applied guaranteed the isolation of a pure population of VW-MSCs from Göttingen Minipigs.

Moreover, the present protocol allowed the isolation of endothelial cells from the tunica intima, preserving their viability and phenotype. Our previous protocol did not permit to collect endothelial cells (EC), but damaged them after excessive vessel manipulation and enzymatic digestion times, while mpAECs obtained with this new protocol were viable and exhibited the endothelial peculiar feature to form a capillary-like network if cultured on an extracellular matrix, confirming what was previously shown for pAECs [74].

## 5. Conclusions

In this paper, we described for the first time the isolation of mesenchymal stem cells from the thoracic aorta of Göttingen Minipigs by using an improved method that preserves the endothelial cell population. The ability to isolate both endothelial cells and vascular mesenchymal stem cells from the same vessel will be useful in planning in vitro 3D heterotypic experiments for studying the contribution of these cellular populations and the cell–cell interactions in physiological and pathological vascular remodeling.

## Figures and Tables

**Table 1:** List of antibodies used for immunofluorescence (IF) and flow cytometry (FC) analyses.

Antibody	P. Number	Species	Supplier	Dilution
*Primary*				
CD45-APC	K252-1E4	mouse	AbD Serotec	10 µL/10^6^ cells
CD90-APC	Ab139364	mouse	Abcam	10 µL/10^6^ cells
CD105-FITC	Ab53318	mouse	Abcam	20 µL/10^6^ cells
CD56-PE	304606	mouse	Biolegend	10 µL/10^6^ cells
CD44-PerPC	103036	rat	Biolegend	10 µL/10^6^ cells
CD34 unconjugated	Ab81289	rabbit	Abcam	1:60
CD31-unconjugated	MCA1746	mouse	AbD Serotec	1:50
VE-Cadherin	MCA1748GA	rat	AbD Serotec	1:25
*Secondary*				
Anti-Rabbit-PE	Ab97070	goat	Abcam	1:200
Anti-Mouse-FITC	F4143	goat	Sigma-Aldrich	1:100
Anti-rat-FITC	F1763	rabbit	Sigma-Aldrich	1:200

## Data Availability

The data presented in this study are available on request from the corresponding author.

## Supplementary Materials

The following supporting information can be downloaded at: https://www.mdpi.com/article/10.3390/ani13162601/s1, Figure S1: Representative images of mpVW-MSCs from the three primary cell lines (MG9, MG11, MG12 respectively). Scale bar 100 µm.
