# Supplementary material for: Isolation of Vascular Wall Mesenchymal Stem Cells from the Thoracic Aorta of Adult Göttingen Minipigs: A New Protocol for the Simultaneous Endothelial Cell Collection
Source: Animals (Basel). 2023 Aug 12;13(16):2601. doi: 10.3390/ani13162601 (PMC10451532; doi:10.3390/ani13162601)
Supplement: Supplementary file 1 [file animals-13-02601-s001.zip › animals-2540585-supplementary.pdf]

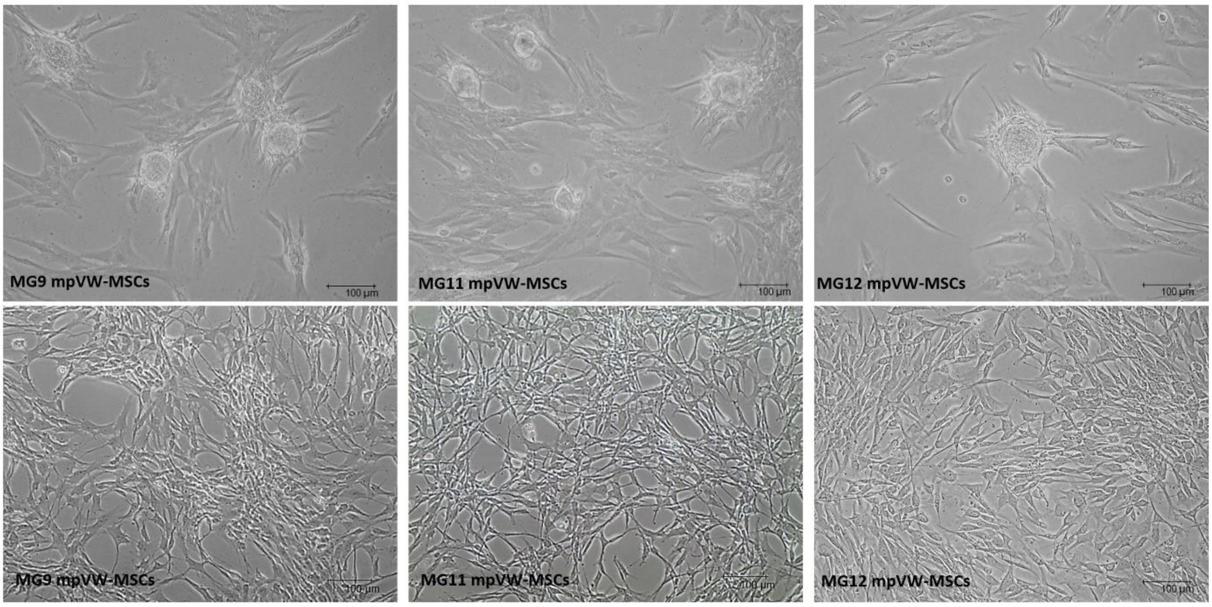

Figure S1. Representative images of mpVW-MSCs from the three primary cell lines (MG9, MG11, MG12 respectively). Scale bar 100 µm.
